# Deep Learning-based Classification of Apical Hypertrophic Cardiomyopathy using Left Ventricular Opacification and Enhanced LV Apex Imaging

**DOI:** 10.2174/0115734056450679260721035709

**Published:** 2026-08-05

**Authors:** P. Venkatesan, A. Rajeswari, N.R. Shanker

**Affiliations:** 1 Department of Computer Science and Engineering, Vel Tech Multi Tech Dr. Rangarajan Dr. Sakunthala Engineering College, Chennai, India; 2 Department of Computer Science and Engineering, Velammal Engineering College, Chennai, India; 3 Department of Computer Science and Engineering, Sriram Engineering College, Chennai, India

**Keywords:** Left ventricular opacification, Apical hypertrophic cardiomyopathy, Gas microbubble, Reverberation artifact, Left ventricular apex

## Abstract

**Introduction:**

Accurate classification of apical hypertrophic cardiomyopathy (ApHCM) subtypes is challenging due to morphological variability and overlapping phenotypes. Conventional echocardiography provides limited visualization of the apex. Artifacts induced during left ventricular opacification (LVO) complicate diagnostic interpretation. A deep learning-based framework enhances image quality and improves subtype classification.

**Materials and Methods:**

In this work, a deep learning-based framework is used for ApHCM subtype classification. Apical four-chamber end-diastolic frames from 3,200 individual patients were extracted from the EchoNet-Dynamic Dataset. Two cardiology experts manually annotated images into pure ApHCM (P-ApHCM), relative ApHCM (R-ApHCM), mixed ApHCM (M-ApHCM), and normal classes, based on apical wall thickness and morphological characteristics, using a computer vision annotation tool. A deep learning pipeline integrated multilevel graph-based adaptive particle swarm optimization with a deep denoised convolutional neural network (MG-APSO-DnCNN) to suppress reverberation and clutter artifacts from LVO echocardiograms. Enhanced images were then segmented using a U-Net-based levelset model to delineate the left ventricular (LV) apex. Morphological and LV wall features were extracted from the segmented region, and a graph isomorphism network (GIN) was trained to capture both local hypertrophic patterns and global ventricular morphology for subtype classification. The framework was designed to distinguish among pure ApHCM, relative ApHCM, and mixed ApHCM.

**Results:**

The framework achieved a classification accuracy of 96.2%, with a precision, recall, and F1-score of approximately 95%. Cross-validation results indicate stable performance (95.8% ± 0.4%, *p* < 0.001), and ablation experiments confirmed the contribution of each pipeline component.

**Discussion:**

By combining denoising, segmentation, and graph-based learning, the framework addressed limitations caused by LVO artifacts and improved the recognition of subtle ApHCM subtypes. These results demonstrate the clinical potential of integrating morphological feature extraction with deep learning in echocardiography.

**Conclusion:**

The framework integrating MG-APSO-DnCNN and GIN enables accurate and robust ApHCM subtype classification, supporting cardiologists in early diagnosis, patient risk stratification, and treatment planning.

## 1 INTRODUCTION

Apical hypertrophic cardiomyopathy (ApHCM) is characterized by localized thickening of the left ventricular (LV) apex [1-4]. The identification and subtype differentiation, including pure, relative, and mixed ApHCM, are essential for risk stratification and clinical management. However, diagnosis is challenging because of overlapping morphological characteristics and subtle structural variations, particularly in early and borderline cases [5, 6].

Echocardiography is the primary imaging modality for cardiac assessment due to its real-time capability, accessibility, and cost-effectiveness [7, 8]. Its reliability in ApHCM evaluation is limited by image artifacts, operator dependency, and suboptimal visualization of the LV apex in apical views [9, 10]. Variability in acquisition angles and foreshortening effects complicate accurate measurement of wall thickness and ventricular geometry, leading to inconsistencies in clinical interpretation [11].

Conventional diagnostic approaches depend on manual measurement of LV wall thickness and visual assessment of geometric patterns. These methods are subject to inter-observer variability when differentiating relative ApHCM from normal myocardium and identifying phenotypes with septal extension [12]. The commonly used apical-to-basal thickness ratio threshold introduces ambiguity, as values near the diagnostic boundary correspond to both early-stage disease and physiological variation [13].

Advances in medical imaging have been integrated with deep learning techniques to automate cardiac segmentation and classification. Convolutional neural networks such as U-Net-based architectures have shown strong performance in delineating LV structures from echocardiographic images [14, 15]. However, these models capture global appearance features and struggle in regions with weak boundaries or low contrast, such as the LV apex. Existing techniques lack explicit anatomical constraints, which result in segmentation leakage and reduced robustness in challenging imaging conditions [16].

To address these limitations, hybrid approaches integrating deep learning with variational and geometric modelling have been proposed. Level set methods provide a framework for evolving contours and incorporating smoothness and boundary regularization to improve segmentation accuracy when combined with data-driven approaches.

Existing methods fail to address clinically ambiguous cases in relative ApHCM. In this subtype, morphological differences are subtle and threshold-dependent. Hence, integrated frameworks are used for accurate segmentation, robust feature representation, and structural understanding.

In this study, we propose a hybrid medical imaging framework that integrates U-Net-based segmentation, level set refinement, and graph-based feature learning for improved classification of ApHCM subtypes. The method enhances boundary delineation and captures morphological relationships for robust classification across four categories, such as pure ApHCM, relative ApHCM, mixed ApHCM, and normal controls. The approach addresses borderline and overlapping cases to enable accurate and interpretable AI-assisted cardiac diagnosis. Figure **1** shows the classification of ApHCM in contrast-enhanced LVO.

Table **1** presents the mapping of the research problem, gap, research questions, and contributions.

The paper is organized as follows: Section 2, Related work, describes the existing techniques for ApHCM classification using deep learning approaches based on apical wall thickness, left ventricular (LV) morphology, and strain analysis. Section 3, Methodology, describes the proposed framework for classifying different types of ApHCM based on shape and morphological features, including aneurysm outpouching from the left ventricular wall. Sections 4 and 5, Results and Discussion, presents classification performance and comparative analysis of the proposed framework. Last section concludes the paper with key findings and directions for future work.

## 2 RELATED WORK

Table **2** summarizes ApHCM detection techniques and their advantages and disadvantages assessed based on LV wall parameters, including LV wall thickness in the apical region, strain parameters, and the systolic and diastolic function of the apex.

## 3 METHODOLOGY

The thickening of the apex raises systolic pressure and wall stress in that area. Consequently, thinner regions bulge toward the apex during heart contraction. Figure **2** shows the architecture for classification of different types of ApHCM using a deep learning algorithm. LVO-based echocardiogram images improve delineation of the endocardial border delineation and the LV apex region.

However, the image quality is poor because the gas microbubble ultrasound enhancing agent creates thick, cloud-like bubbles. These bubbles interact with ultrasound waves, generating clutter and reverberation artifacts. Reverberation artifacts produce similar patterns, while clutter artifacts create bright spots, which affect the LV wall region in patients with obesity, lung disease, ventilatory support, and chest wall deformities.

The combined MG-APSO-DnCNN enhances the LVO echocardiogram image at the apex and aneurysm region by preserving the structure of the endocardial border at the LV apex and aneurysm regions, along with geometric features, by reducing clutter and reverberation artifacts. The CNN-based level-set segmentation algorithm segments the boundaries of the LV wall, regulates smoothing boundaries at the edges without discontinuity, and preserves the pixel-level features. Next, shape and morphological features are extracted from the segmented LV wall at the apex. Finally, the features are classified using GIN, which captures regional changes among different LV regions by capturing topological patterns across complex LV regions. Figure **3** shows the process flow diagram of the proposed architecture for classifying different ApHCM subtypes.

### 3.1 Dataset Description

The EchoNet-Dynamic dataset contains 10,030 apical 4-chamber echocardiographic videos from adult individuals. These videos were manually annotated with end-systolic and end-diastolic frames as described in EchoNet Dynamic. The dataset does not contain annotations for Apical Hypertrophic Cardiomyopathy. A manual annotation protocol was conducted. Two cardiologists (Dr.Aarthi and Dr.Alex M. George) selected 3,200 end-diastolic apical 4-chamber echocardiographic frames. These frames were annotated using the CVAT annotation tool. The left ventricular apex, endocardial border, and epicardial border were identified, and myocardial wall thickness was measured at end-diastole. Based on these measurements, images were categorized into four classes: pure ApHCM (*n*=782), relative ApHCM (*n*=984), mixed ApHCM (*n*=939), and normal (*n*=495). Disagreements between the two observers were resolved by a senior cardiologist, and interobserver agreement was assessed using Cohen's κ coefficient.

To avoid leakage, the dataset was partitioned into training (*n* = 2,275), validation (*n* = 469), and hold-out test (*n* = 456) using stratified sampling. Five-fold cross-validation was performed on the training set. The test set was used for final evaluation.

Table **3** represents the class distribution of the ApHCM dataset and interobserver agreement among classes. Table **4** describes the inclusion and exclusion criteria.

### 3.2 Apical Left Ventricular Wall Region Image Enhancement using a Multilevel Graph- Based Adaptive Particle Swarm-Optimized DnCNN (MG-APSO-DnCNN)

LVO serves as a contrast-enhanced ultrasound imaging agent that enhances the LV chamber and improves endocardial border delineation. This contrast agent enables accurate assessment of LV volumes, ejection fraction, regional wall motion, and structural abnormalities through blood pool opacification. LVO improves visualization of multiple LV segments and facilitates the identification of thrombi, tumors, and aneurysms.

Although LVO-based images improve LV visualization, they may also contain near-field artifacts, which occur close to the transducer and affect the apex region, as well as clutter and reverberation artifacts, arising from microbubble echoes, which generate false signals near the LV wall boundary (Fig. **4**). These artifacts can produce false signals in the apical wall region, potentially leading to misdiagnosis of thrombi or apical aneurysms and complicating the identification of apical morphological variants and reducing diagnostic precision.

To enhance image quality, MG-APSO-DnCNN was applied. The DnCNN architecture removes Gaussian noise from echocardiogram images but is less effective at removing clutter and reverberation artifacts. The MG-APSO-DnCNN model preserves critical boundaries and structural details, including apical wall thickness variations and the characteristic spade- shaped morphology, through a multilevel graph of interconnected pixels. The APSO-tuned DnCNN targets patient-specific artifact patterns in echocardiographic images, including swirling artifacts in individual cases of dilated left ventricles, signal attenuation and acoustic shadowing due to variations in LV geometry and contrast agent dosing. Additionally, blooming artifacts arise from the intrinsic acoustic properties of cardiac tissue. Figure **5** illustrates apical LV wall enhancement achieved using MG-APSO-DnCNN.

The mathematical model of the MG-APSO-DnCNN is shown in eq. (**1**).


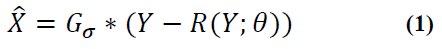


In eq. (**1**), (*G_σ_*) denotes the Gaussian Smoothing operator. ‘Y’ represents the ultrasound image. *R*(*Y*; *θ*) indicates the residual noise estimate of the image, and 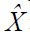 is the denoised image. The mean squared error is minimized through optimizing the number of layers and the size of the convolutional filters, removing the noise and preserving the anatomical details of the LV wall region. The objective function to be minimized is given in eq. (**2**).


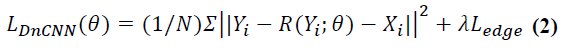


In eq. (**2**), *X_i_* is defined as the clean image, N is the number of samples, *and L_edge_* is a preservation loss. 
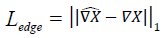
 and *λ* is the edge weight operator. ∇ denotes the Sobel gradient operator.

To remove the pattern-specific noise from LVO-based echocardiogram images, DnCNN layers were refined using the APSO algorithm. The APSO algorithm optimizes the number of convolution layers and the number of filters within the DnCNN. An insufficient or excessive number of layers and filters suppresses noise while compromising image features. APSO-DnCNN enhances the quality of the LV apical region and LV wall segments. The APSO optimization is expressed in eq. (**3**).

Each particle is defined as *Particle: p_i_* = [*L_i_*, *F_i_*], where *L_i_* is the number of layers and *F_i_* is the filter size.

The velocity update is defined in equation (**3**).


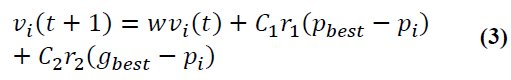


In eq. (**3**), *v_i_* is the initial velocity at time ‘t’, *w* is the inertia weight. *C*_1_ and *C*_2_ are the cognitive and social acceleration coefficients. *r*_1_, *r*_2_ are random values uniformly distributed in (0,1), *p_best_* is the personal best position. *g_best_* is the global best position. ‘w’ is inertia weight.

*c*_1_ and *c*_2_ are cognitive and social acceleration constants that guide particles toward global best positions. The position update rule is given by *p_i_*(*t* + 1) = *p_i_*(*t*) + *v_i_*(*t* + 1), which balances exploration and exploitation to prevent premature. To model structural relationships among the LV wall regions, such as apical, basal, and mid-ventricular areas, a multilevel graph-based structure-aware attention network was used. This network captured the relationships among anatomical regions through superpixel-based segmentation, and node features were computed from texture descriptors to ensure continuous, sharp boundaries along the LV wall. The multilevel graph-based APSO-DnCNN was used to preserve the fine structural detail of the LV wall region in echocardiographic images and enhance image quality. The multilevel graph structure of MG-APSO-DnCNN is defined in eq. (**4**).


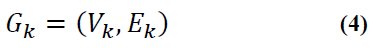


In eq. (**4**), *G_k_* represents the multilevel graph and *V_k_* indicates the set of vertices that corresponds to the image regions based on the texture features of LV. *E_k_* denotes the set of edges defined by feature similarity between spatially adjacent superpixels. Hence, the optimal DnCNN configuration is defined in eq. (**5**).


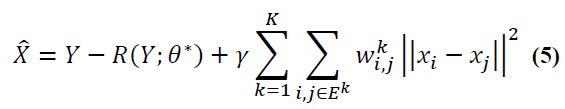


In eq. (**5**), θ* is defined as the APSO-optimized parameter vector and *w^k^_i,j_* is the edge weight between nodes i and j at graph level k, γ is the graph regularization parameter. 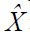 is a denoised image and *Y* is a noisy echocardiogram image, *R*(*Y*;θ*) is a noisy echocardiographic image, and θ is the network parameter (L, F).

Table **5** shows the configuration details of the MG-APSO-DnCNN. Table **6** lists the parameters used for MG-APSO-DnCNN. Figure **6** shows the convergence curve of MG-APSO-DnCNN. Figure **7** displays the enhanced apical region using MG-APSO-DnCNN.

Ground-truth reference images for PSNR and SSIM were generated in a controlled environment. A cardiologist selected a clean echocardiographic image with minimal noise. Clutter and reverberation artifacts were simulated using multiplicative speckle noise and Gaussian electronic noise. The MG-APSO-DnCNN method was then applied to remove artifacts, and quantitative metrics were calculated between the reference and processed images.

### 3.3 Extraction of LV wall using U-Net-Level Set Segmentation

Accurate classification of ApHCM depends on criteria including regional wall thickness of ≥15 mm and maximal apical-to-posterior wall thickness of ≥1.5. LV wall segmentation has several advantages such as: (i) It increases the diagnostic accuracy of ApHCM classification by differentiating anatomical LV wall regions; (ii) It enables analysis of structural changes in the LV apical region, such as apical aneurysm; and (iii) It helps differentiate ApHCM from other heart diseases, such as hypertensive heart disease. Figure **8** illustrates the segmentation process of the LV wall for ApHCM classification using U-Net-levelset segmentation. U-Net-based segmentation requires a large annotated dataset, which can reduce segmentation accuracy incases incolving the complex shape of LV myocardium. Level set segmentation requires an initial contour and often struggles with weak boundary definition. Therefore, to accurately preserve the anatomical LV apical region with an aneurysm, a combination of U-Net and level set segmentation was necessary for precise LV wall segmentation. The method utilized the U-Net model to construct the initial contour, while the level set method refined this contour using prior shape boundaries and delineated the epicardial and endocardial boundaries. The combined approach effectively preserved the geometric shape of the LV wall. Table **7** shows the U-Net-based architecture of LV wall segmentation. Figure **8** describes the segmentation process of the LV wall using U-Net and level-set segmentation.

To initialize the level-set contour, the mask was converted into a signed distance function. The level-set algorithm then refined the contour over 50-100 iterations by minimizing the energy functional. To address the segmentation errors in weak-edge regions of echocardiographic images, an edge-stopping function and a shape prior U-Net-derived shape prior probability map were integrated into the level-set formulation. The implementation steps for equation (6) are provided in Supplementary File **S1**. The fusion mechanism between U-Net and the level-set framework was used for information flow and parameter balancing. In the proposed approach, the U-Net output was used as a probability map *P(x)* in [0,^1^] that indicates the pixel-wise confidence of the LV wall. This probability map was used to initialize the level set function and serve as a shape prior during contour evolution. Hence, the U-Net probability map moved the contour towards the LV region, and the edge-stopping function avoided leakage at noisy edge boundaries. A higher weight (*λ*) was assigned for convergence towards the LV shape, and the edge weight (*η*) was incrementally increased during iterations to refine the boundary. The smoothness parameter (*μ*) regulated the contour and suppressed sensitivity to weak edges in the myocardial region. The region-based terms were dynamically updated using the mean intensities of the evolving contour. Hence, the fusion of U-Net with the level set provided global structural and local geometric boundary precision in LV wall segmentation under weak-edge conditions.

The mathematical model of combined U-Net-based level set segmentation is defined in eq. (**6**).


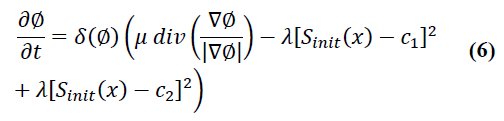


In eq. (**6**), Ø is defined as the level set function representing the contour. 
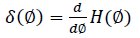
 represents the Dirac delta function and *H*(Ø) is the heavy side function, which localizes the contour near the segmented boundary. *µ div* 
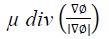
 indicates the curvature and regulates the discontinuities of the edges around the LV apex and the aneurysmal regions-features associated with thin LV myocardium. *S_init_*(*x*) represents the initial contour. *c*_1,_ *c*_2_ represent the foreground and background mean intensities. *c*_1_ moves the contour inside towards the boundary based on the curvature *S_init_*(*x*) and *c*_2_ moves the contour outside the boundary of the initial contour *S_init_*(*x*). Table **8** describes the optimal U-Net and level set parameters used for segmentation. Table **9** shows the values of parameters used to segment the LV wall. Table describes the optimal U-Net and level set parameters.

### 3.4 Classification of ApHCM using a GIN Network through LV Wall Morphological Features

The classification of ApHCM depends on left ventricular (LV) wall morphological features, including apical and basal characteristics, the apical-to-basal ratio, and spade-shape related features such as eccentricity, solidity, concavity, and maximum and minimum wall thickness. Hence, ApHCM subtypes are classified using a GIN Network as illustrated in Fig. (**9**). Before the graph construction, the morphological features were standardized using Z-score normalization applied to geometric descriptors and wall thickness measurements. The detailed feature normalization and graph construction processes are included in Supplementary File **S1**.

These morphological features were converted into a graph in which each feature acted as a node, and the edges represented anatomical relationships between nodes based on statistical correlations with imaging structural links. Each node was connected to all other nodes with edge weights reflecting the degree of interaction among morphological features. In the input node feature graph, blue indicates below-mean values, while red indicates above-mean values.

The input node feature matrix is expressed in eq. (**7**):


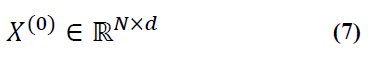


In eq. (**7**), N is the number of nodes and ‘d’ is the feature dimensionality. Hence, X is a set of feature vectors {Eccentricity, Solidity, Concavity, Max wall thickness, Min wall thickness, Apical wall thickness, Basal Wall thickness, Posterior Wall thickness, Apical to Basal Ratio}

The input graph is represented in the adjacency matrix A with N×N

The nodes were pre-processed using equation (**8**):


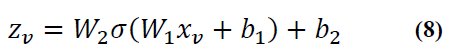


In eq. (**8**), *W*_1_ and *W*_2_ represent the learned weights of the linear layer that transforms the aggregated output into a hidden representation. *σ* indicates the Rectified Linear Unit, which learns the complex pattern among the morphological features of ApHCM hypertrophies and subtypes of ApHCM. *b*_1_ and *b*_2_ represent the biases. Hence, the final node feature embeddings were generated using eq. (**8**).

The node embedding features are represented in eq. (**9**):


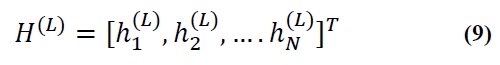


In eq. (9), *h*^(^*^L^*^)^*_v_* is the final embedding feature vector after GIN convolution, capturing local and neighbourhood-level information for each cardiac feature.

The global mean pooling *h_G_* is represented in eq. (**10**):


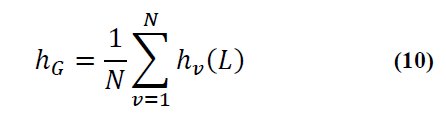


In eq. (**10**), N is the total number of nodes (*N= 9*). *h_G_* is the single graph-level feature vector encoding the overall cardiac structure of the patient. Node features were standardized using Z-score normalization computed from the training set and clipped to the range [−5, 5] to ensure robustness against outliers. Edge weights were defined using a Gaussian kernel combining spatial proximity, feature similarity, and an anatomical adjacency mask to preserve physiologically meaningful relationships between LV regions.

Table **10** represents the overall architecture of the GIN network for ApHCM subtype classification. Table **11** lists the training parameters of the GIN network.

## 4 RESULTS

The performance of the ApHCM classification was evaluated using the metrics shown in eqs. (**11**-**14**).


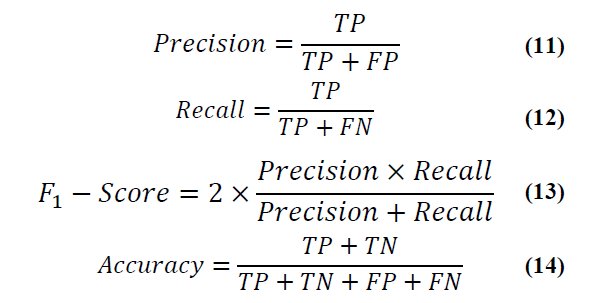


From eqs. (**11**-**14**)

True Positive (TP): The patient actually has ApHCM, and the model correctly predicts ApHCM.

False Positive (FP): The patient does not have ApHCM, but the model incorrectly predicts ApHCM.

True Negative (TN): The patient does not have ApHCM, and the model correctly predicts a non-ApHCM

False Negative (FN): The patient actually has ApHCM, but the model incorrectly predicts a non-ApHCM case.

### 4.1 Performance Analysis of ApHCM Classification using Different Graph-Based Deep Learning Models

Figure **10a** illustrates the training and testing accuracy curves, and Fig. (**10b**) represents the training and testing loss curves of various graph-based deep learning models in ApHCM classification. In this study, ApHCM classification relied on the relationship between the morphological and functional parameters of the left ventricle. In ApHCM and its subtypes, there exists a correlation between the wall thickening in one region and geometric changes in another region. Graph-based models capture structural relationships, preserve clinical topology, and adapt to multimodal data without loss of spatial fidelity. The GIN model employs the Weisfeiler-Lehman(WL) test to determine whether two graphs are isomorphic. In the graph, each node is represented by a 9-dimensional normalized feature vector, such as eccentricity, solidity, concavity, maximal wall thickness, minimal wall thickness, apical wall thickness, basal wall thickness, posterior wall thickness, and the apex-to-basal thickness ratio. Nodes correspond to LV regions such as the apical, mid, and basal segments, and edges represent inter-regional spatial adjacency. Through WL-based feature aggregation, the model captured structural relationships between the apex and the surrounding ventricular region to classify ApHCM. The detailed description of the WL test is provided in Supplementary **S1**.

Figure **11** displays the ROC curves for pure ApHCM, relative ApHCM, mixed ApHCM, and normal hearts using different graph-based deep learning models. GIN achieved the highest ROC AUC (0.97-0.98) in detecting both apical and non-apical morphologies, demonstrating its capacity to capture fine-grained structural features, such as changes in apical wall thickening at the aneurysm and variations in endocardial contour, which facilitated subtype discrimination. Table **12** presents the AUC and ROC values for each ApHCM class, along with their corresponding morphological features, to differentiate between ApHCM and non-ApHCM using various graph-based deep learning models.

Table **13** presents classification metrics for each class along with their morphological interpretations. For pure ApHCM, GIN achieved a precision of 0.89, a recall of 0.88, and an F1-score of 0.88, identifying apex-based hypertrophy with minimal spread into mid or basal walls. In relative ApHCM, precision (0.89) and recall (0.90) were well balanced, indicating feature-level detection of apex-basal morphological relationships. For mixed ApHCM, precision, recall, and F1-score all reached 0.90; GIN detected complex hypertrophy patterns, including extension from the apex into the mid or basal segments. In normal cases, both precision and recall were 0.90, illustrating the capability of the GIN-ApHCM model to accurately identify normal ventricular morphology.

Figure **12** illustrates the echocardiography-based morphological variations in the ApHCM and its subtype classification. In Fig. (**12a**), the boxplot shows the correctly classified apical wall thickness, with misclassified cases indicating that pure apical wall thickness exceeds 18-21 mm, and pure ApHCM shows a thick apex. Relative ApHCM has a lower apical thickness range of 13-15 mm. Fig. (**12b**) presents kernel density plots that display the correct and misclassified apical/basal ratios in wall thickening, indicating more apical thickening than basal. Misclassified cases around 1.0 values show less apical and more basal involvement values, complicating the differentiation of non-apical hypertrophy. Fig. (**12c**) shows density plots of the spade-shaped LV-cavity index, which correctly shows the density peaks at high spade-shaped values greater than 0.6. Fig. (**12d**) illustrates the confusion matrix for ApHCM classification using the GIN network.

The misclassification probabilities with 95% confidence intervals show that relative ApHCM has the highest uncertainty (0.148, 95% CI: 0.130-0.166) due to phenotypic morphological overlap. Table **14** displays the Qualitative analysis of misclassified cases.

The model performance was evaluated using 5-fold cross-validation and a hold-out test set to ensure robustness and generalization, as shown in Table **15**. The proposed method achieved 95.8% accuracy in 5-fold cross-validation (±0.4% CI), which indicates strong predictive capability. Statistical significance analysis indicates that the proposed model performed significantly well (*p*<0.001). Table **16** shows the 5-fold cross-validation for each training and validation set.

Table **17** shows that the proposed GIN-ApHCM model achieves an accuracy of reported 96% due to its WL-based neighbourhood aggregation strategy. The GIN-ApHCM model encodes the relationships among the apex, septum, and basal wall using nodes and edges, combining local and global structural information from the apical segment and the endocardial LV region. GIN-ApHCM integrates myocardial wall thickness, spade-shaped transformations, and segmental morphology into graph-structured data and captures the complex interrelationships in ApHCM more effectively than traditional deep learning and machine learning models.

## 5 DISCUSSIONS

Apical Hypertrophic Cardiomyopathy (ApHCM) results from the thickening of the heart muscle at the left ventricular (LV) apex. This thickening reduces the efficiency of cardiac pumping and increases the risk of arrhythmias, heart failure, and sudden cardiac death. The proposed GIN-ApHCM model achieved an accuracy of 96.2%, demonstrating high sensitivity to apex-dominant hypertrophy patterns involving minimal basal or mid-wall extension. The model also achieved an accuracy of 95% for relative ApHCM, indicating its effectiveness in recognizing transitional morphological features that involve both apical and basal segments while minimizing the false negative rate relative to pure ApHCM detection. Mixed ApHCM reached an accuracy of 96%. Hence, GIN captured complex, multi-segmental hypertrophy patterns extending from the apex to the mid and basal regions. Table **18** shows the ablation study on the GIN-ApHCM model for ApHCM and its subtype classification.

Traditional methods of identifying ApHCM often result in reduced accuracy in classification due to limited visualization, morphological overlap, insensitivity to complications, and inadequate assessment of LV wall function.

Figure **13** shows the feature correlation analysis of morphological parameters of the LV wall and the LV apex region. The apical and maximal wall thickness correlation is 0.96, indicating that the ApHCM classification depends on the apex region. Eccentricity shows a correlation (r = 0.83), indicating the significance of apex geometry in the LV wall contour. The apical-basal ratio and apical thickness (r = 0.78), as well as concavity and apical thickness (r = 0.66), show moderate correlations between geometric deformation and localized thickening. Basal wall thickness shows a negative correlation with the apical-basal ratio (r = −0.36), indicating an inverse relationship between the apex and base in certain subtypes.

The feasibility of clinical deployment of the proposed MG-APSO-DnCNN + GIN framework has been evaluated in terms of inference efficiency and integration capability. The model was implemented using PyTorch and tested on a workstation equipped with an NVIDIA RTX 3090 GPU (24 GB VRAM) and an Intel i9 processor. The average inference time for a single LVO echocardiographic frame encompassing denoising, segmentation, feature extraction, and classification was 0.18-0.25 seconds, enabling real-time analysis. The MG-APSO optimization was performed offline during training. Table **19** shows the Inference time analysis during clinical deployment of ApHCM subtypes.

Figure **14** illustrates the association between the predicted probabilities from the proposed GIN-ApHCM model and the observed proportion of high-risk left ventricular hypertrophy (LVH) cases, as ApHCM is a specific type of LVH. The figure demonstrates the model's strong capability in clinical risk stratification. As the predicted probability bins values increase from 0.258 to 0.949, the proportion of high-risk LVH cases also rises. Therefore, the proposed model effectively discriminates between lower- and higher-risk patients and supports the identification of early intervention strategies and resource allocation in clinical settings. Table **20** illustrates the sample morphological features of different ApHCM subtypes.

## 6 LIMITATIONS

The model was trained on a limited dataset that does not account for demographic and echocardiographic variability. This limitation may reduce diagnostic accuracy due to variability in ultrasound probe settings and contrast agent (microbubble) dosing protocols. Ground truth labelling is challenging because of morphological variability between pure and mixed ApHCM phenotypes. The dataset does not contain paired CMR data for direct comparison. The current model focuses on structural features from end-diastolic echocardiographic frames and does not incorporate dynamic cardiac motion features, precluding assessment of dynamic LVOT obstruction, a clinically significant ApHCM characteristic. The extracted morphological features were normalized to reduce inter-patient scale differences; however, normalization based on body surface area was not explicitly considered, which may introduce systematic bias in classification across patients of differing body habitus. Furthermore, PSNR and SSIM evaluation relied on synthetically simulated artifacts rather than clinically annotated artifact maps, which represents a methodological limitation of the enhancement module.

## CONCLUSION

The classification of ApHCM is challenging because of morphological variation and overlapping phenotypes of LV myocardium structure. Thickening of the wall leads to the formation of aneurysms in some cases, complicating the differentiation of thrombus formation. Consequently, small changes in the LV apex region can result in sudden cardiac death and heart failure. Therefore, early identification of ApHCM increases the patient survival rate. A hybrid framework that combines deep convolutional LV region enhancement with a graph-based GIN model utilizes local hypertrophic morphological features and global geometric variability for robust ApHCM classification. This model achieved a classification accuracy of 96.2%, precision of about 95%, recall of about 95%, and an F1 score of about 95%. The integration of AI-based image analysis enhanced the characterization and classification of ApHCM. In future work, the proposed GIN-ApHCM framework will be extended to diverse populations and imaging protocols to enhance generalizability. The model will incorporate echocardiographic video sequences to capture functional cardiac dynamics. In future work, the proposed GIN-ApHCM framework will be extended to diverse populations and imaging protocols to enhance generalizability. The model will incorporate echocardiographic video sequences to capture functional cardiac dynamics. Future iterations will also account for body surface area normalization and incorporate rare variants such as apical aneurysm and thrombus formation with more diverse clinical cases to evaluate model performance in complex scenarios.

## Figures and Tables

**Fig. (1):**
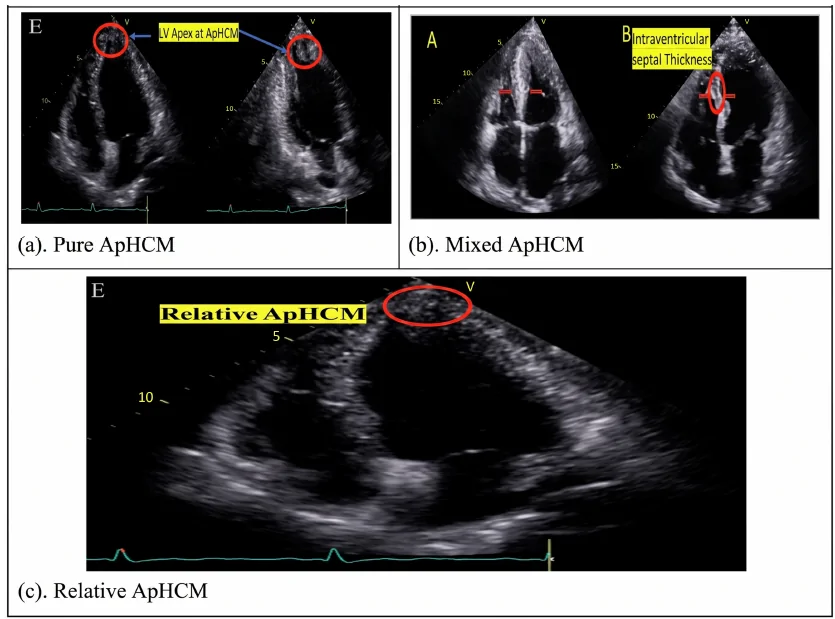
Classification of ApHCM in Contrast-Enhanced LVO. (**a**) Four-chamber and two-chamber views of pure ApHCM. (**b**) Four-chamber view of mixed ApHCM, where wall thickness extends up to the interventricular septum, excluding the apex in the LV, with a diminished spade shape [15]. (**c**) Four-chamber view of relative ApHCM [16].

**Fig. (2):**
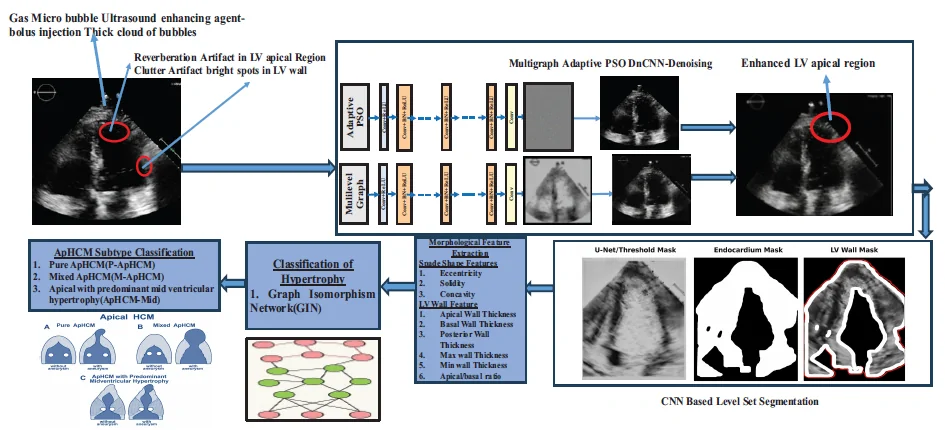
Proposed deep learning architecture for ApHCM subtype classification with LV apex region enhancement from contrast- enhanced LVO image.

**Fig. (3):**
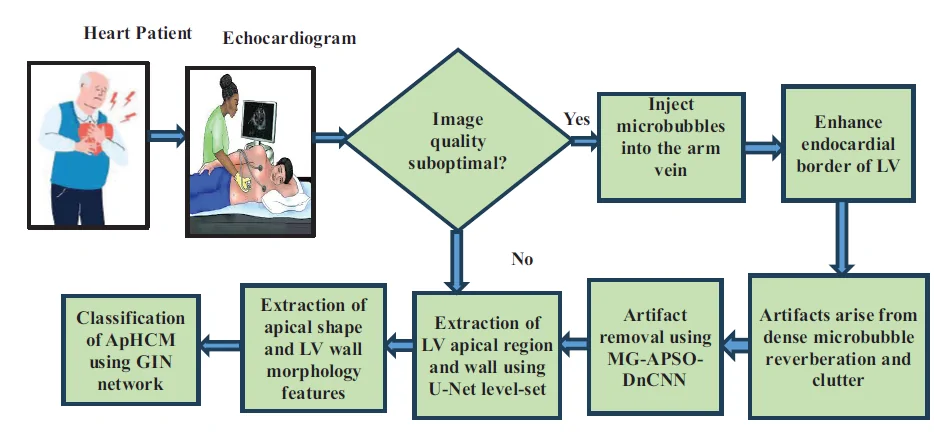
Process flow diagram of the proposed architecture for classifying different types of ApHCM.

**Fig. (4):**
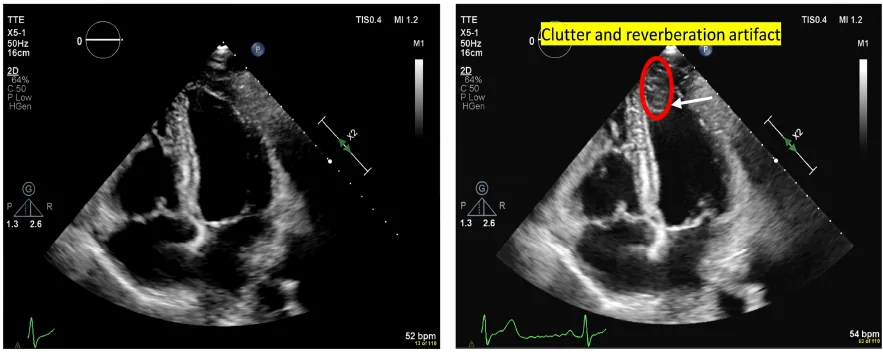
Clutter and reverberation artifact of echocardiogram image.

**Fig. (5):**
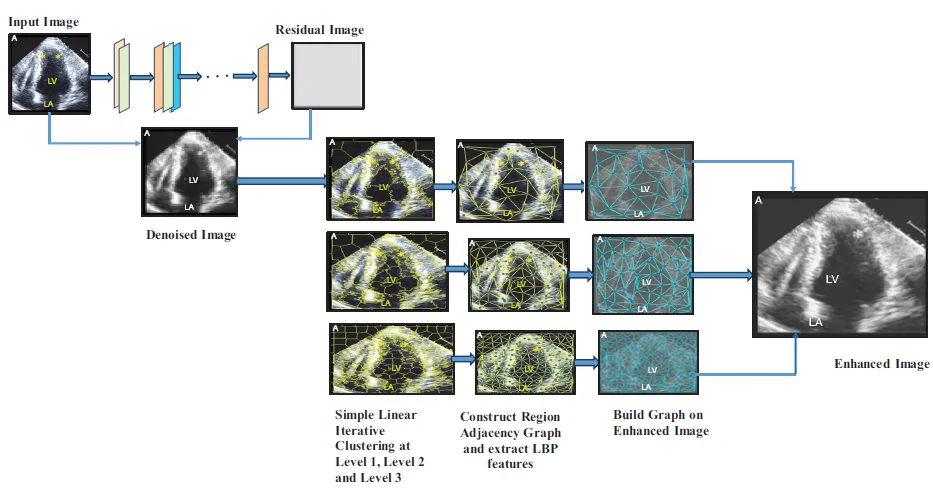
Process of Apical LV enhancement using MG-APSO-DnCNN.

**Fig. (6):**
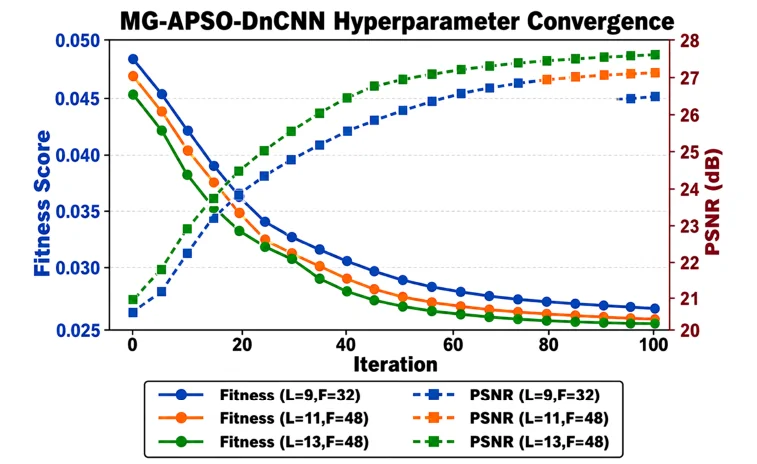
MG-APSO-DnCNN hyperparameter convergence.

**Fig. (7):**
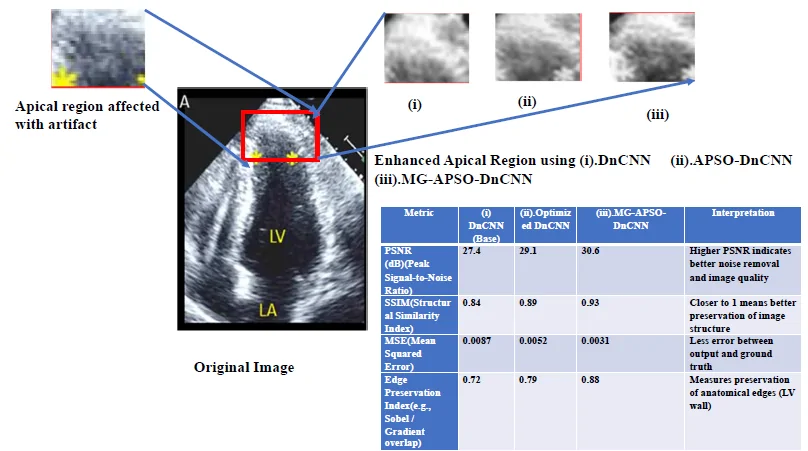
Enhanced apical region using MG-APSO-DnCNN.

**Fig. (8):**
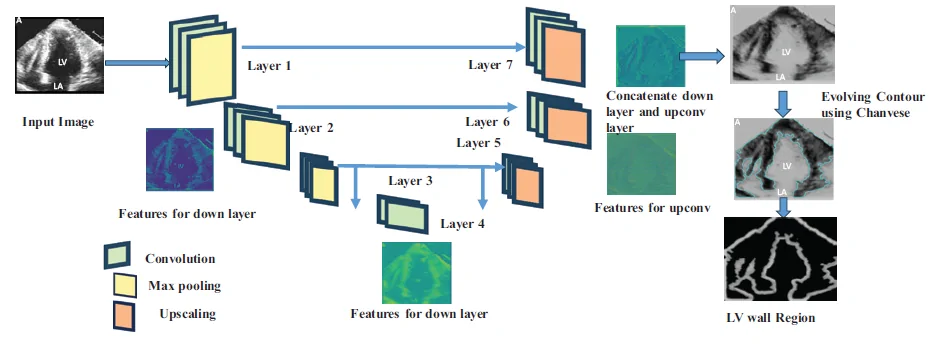
Segmentation process of LV wall using CNN-based level set segmentation.

**Fig. (9):**
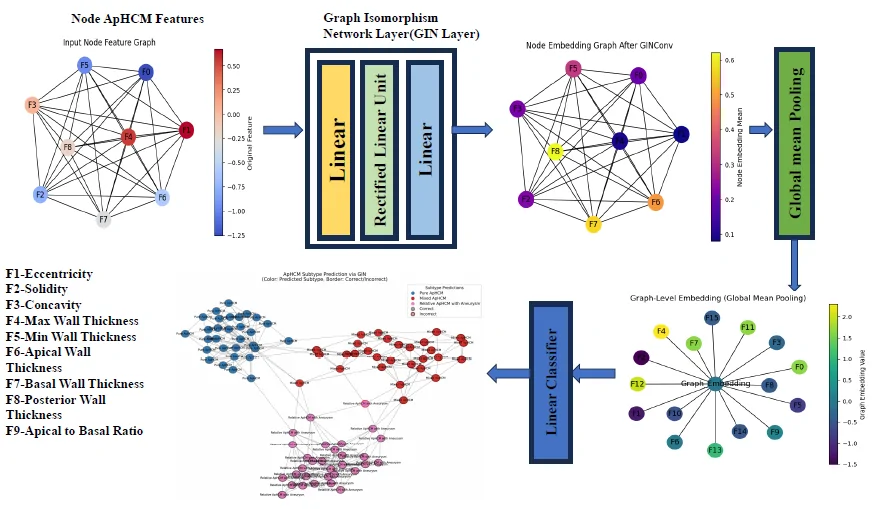
Classification of ApHCM using GIN with morphological features.

**Fig. (10a, b):**
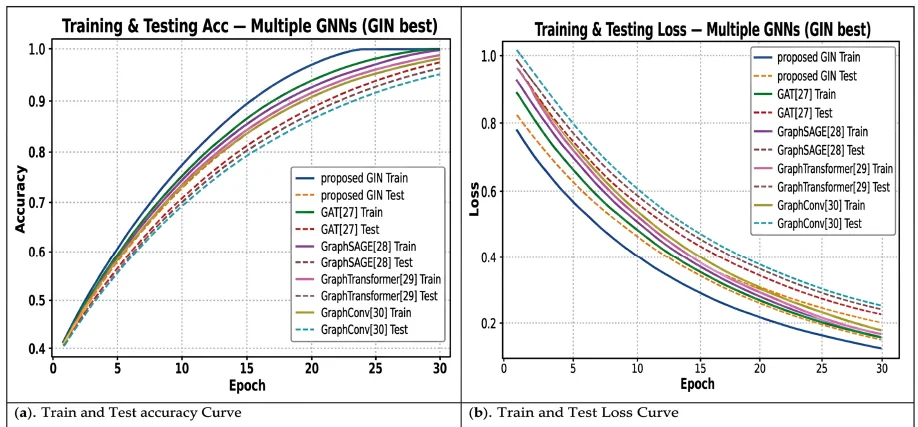
Training and testing loss and accuracy curves.

**Fig. (11):**
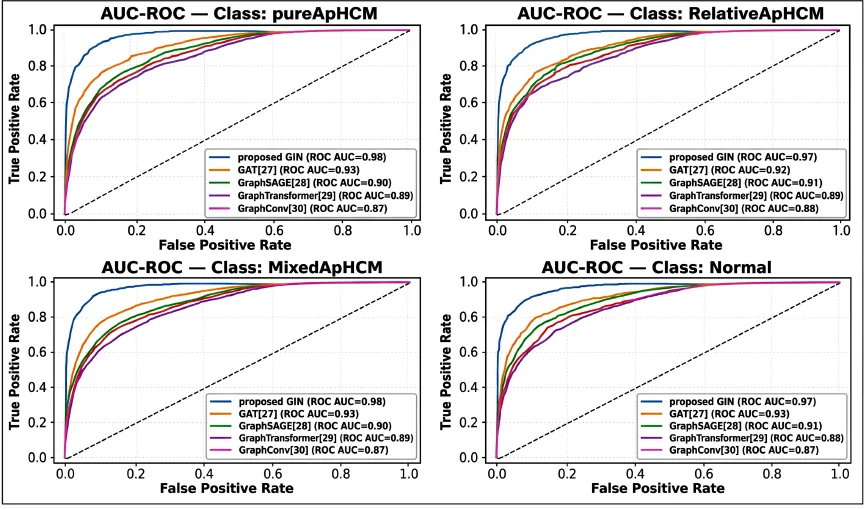
ROC Curve of ApHCM subtype classification using Graph-based deep learning model.

**Fig. (12a-d):**
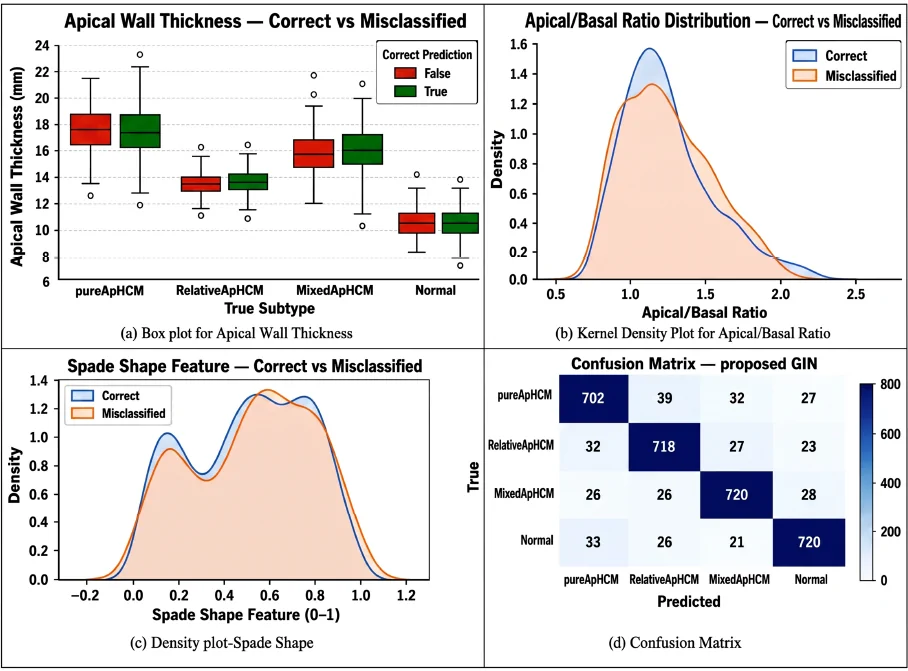
Echocardiographic morphological variation in ApHCM and its subtypes.

**Fig. (13):**
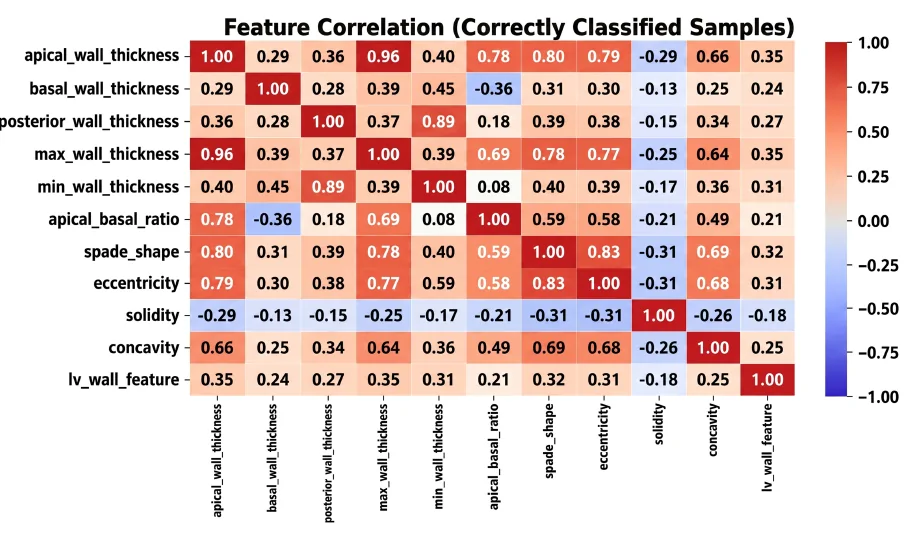
Feature correlation analysis of morphological parameters in ApHCM classification.

**Fig. (14):**
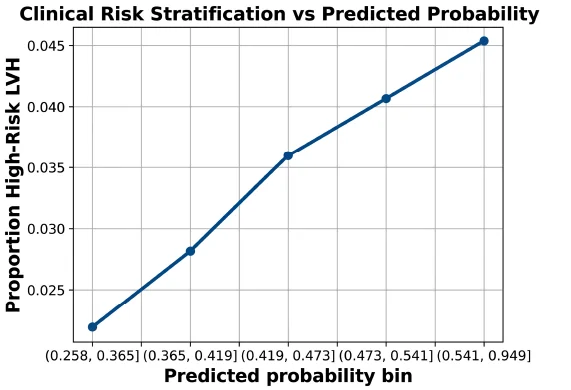
Comparison plot for clinical risk of high and predicted probability of ApHCM classification.

**Table 1:** Mapping research problem, gap, research questions, and contributions.

**Problems and Research Questions**	**Description**	**Proposed Contributions**	**Performance**
**Problem**	LVO clutter and reverberation artifacts obscure the LV apex and mislead thickness assessment.	#1 - MG-APSO-DnCNN (artifact-aware denoising)	Accuracy +2.1%, PSNR 32.4 dB
**Gap**	Prior echo-AI methods ignore apex morphology and inter-region relations and rely on frame-level splits.	#2 - U-Net + Level-Set (precise apex segmentation) and #3 - GIN (graph morphology)	Dice 0.94; F1 0.96
**Question 1**	Do artifacts lower ApHCM classification accuracy?	#1 - Denoising module	H1 validated (*p* < 0.001)
**Question 2**	Does segmentation quality improve subtype classification?	#2 - Segmentation module	H2 validated ( +3.4 %)
**Question 3**	Does combining denoising, segmentation, and morphology outperform single blocks?	**Integrated pipeline (#1 + #2 + #3)**	Overall96.2 % [94.1-98.3 %] CI

**Table 2:** Related work on ApHCM detection techniques.

**Reference**	**Imaging Modality**	**Parameters used for Assessment**	**Advantages**	**Disadvantages**	**Research Gap**
[17]	Echocardiography	Apical wall thickness, LV morphology, functional parameters	Detailed clinical correlation	Requires expertise in advanced imaging interpretation	Need for standardized imaging modalities
[18]	Echocardiography with machine learning	LV wall thickness	Reduces human error	Dependent on quality and quantity of training data	Generalizability and integration in clinical workflow
[19]	Echocardiography, deep learning algorithm	Classification of LV segments by hypertrophy severity	Segment-level analysis	Computationally intensive	Application to diverse populations
[20]	Echocardiography, morphologic analysis	Morphologic and functional characteristics of LV trabeculation	Differentiates LV trabeculation *vs*. apical hypertrophy	Overlap in imaging features	Difficult to differentiate similar phenotypes
[21]	Echocardiography, deep learning	Multiple echocardiographic features, including wall thickness and strain	AI for enhanced imaging analysis	Limited adaptability to real-world clinical challenges	Need for standardized benchmarks
[22]	Echocardiography (Speckle Tracking)	LV apex mechanics, strain parameters, systolic and diastolic function of apex	Detailed functional assessment of LV apex; non-invasive	Requires advanced echo techniques and expertise	Need for correlation with clinical outcomes
[23]	Multimodality imaging review	Anatomical and functional parameters	Usage of advanced imaging modalities	Broad review, lacks ApHCM-focused quantitative data	Challenges in integrating novel imaging biomarkers
[24]	Cardiac MRI, perfusion imaging	Myocardial perfusion parameters	High spatial resolution	Computationally intensive	Limited exploration of therapy impact on perfusion and clinical outcomes
[25, 26]	Echocardiography, deep learning framework	LV wall thickness differentiation, automated classification of hypertrophy etiologies	Reduces operator variability	Needs large and diverse datasets; deep learning interpretability issues	Requires clinical validation across populations

**Table 3:** Class distribution of the ApHCM dataset and interobserver agreement.

**ApHCM Subtype**	**Training**	**Validation**	**Testing**	**Total**	**Actual (Kappa)**	**Agreement Level**
Pure ApHCM	560	112	110	782	0.87	Almost perfect
Relative ApHCM	700	140	144	984	0.79	Substantial
Mixed ApHCM	660	145	134	939	0.82	Almost perfect
Normal (Control)	355	72	68	495	0.91	Almost perfect
**Overall**	**2275**	**469**	**456**	**3200**	**0.85**	Almost perfect

**Table 4:** Inclusion exclusion criteria.

**Category**	**Criteria Type**	**Description**
**Initial dataset**	Data collection	Total echocardiographic videos collected: **10,030**
**Inclusion criteria**	View requirement	Standard apical views (**A4C / A2C**)
	Anatomical visibility	Clear visualization of **LV apex** and **endocardial borders**
	Temporal coverage	Complete **cardiac cycle** coverage
	Image quality	Adequate quality for **morphological analysis**
**Exclusion criteria**	Image quality issues	Poor quality due to **noise, motion artifacts, or shadowing**
	Anatomical limitation	Incomplete LV visualization, especially **apex region**
	Clinical conditions	Presence of other cardiac diseases (*e.g*., **dilated cardiomyopathy, valvular disease**)
	Data redundancy	Duplicate or repeated recordings
**Annotation and verification**	Expert review	Independently reviewed by **two cardiologists**
	Classification	Categorized into: • Pure ApHCM • Relative ApHCM • Mixed ApHCM • Normal (control)
	Conflict resolution	Disagreements resolved by **consensus discussion**
**Final dataset**	Selected cases	**3,200** echocardiographic videos
	Class distribution	Balanced distribution across **four classes**

**Table 5:** Configuration of the proposed MG-APSO-DnCNN.

**Layers**	**Configuration/Equation**	**Description & Role**
Input image	1×64×64	Noisy ultrasound image (with clutter and reverberation)
DnCNN hyperparameters	Number of layers (5,20) Number of filters (32,124)	Number of convolutional layers and filters per layer, optimized by APSO: L=13, F=48
DnCNN layer 1	3×33	Input layer extracting features from noisy image
DnCNN layers 2-12	3×33	Intermediate layers for deep feature learning, repeated L−2 - 12 times
DnCNN layer 13	3×33	Output layer predicting the noise residual
DnCNN output	Enhanced image	Denoised image, input for graph construction
Multilevel graphs	Levels: k=1,2,3 k = 1,2,3 (50, 100, 200 super pixels)	Each level models spatial/texture structure at different scales
Graph nodes	Regions super pixels at level	Represent anatomical regions (*e.g*., LV wall)
Graph edges	Edges	Capture spatial relationships between nodes
Node features	*e.g*., LBP histogram	Used to compute edge weights and smoothness
Best configuration	(13,48)	Found using APSO after multiple fitness evaluations
Final output	Enhanced image	Clutter- and reverberation-free image with preserved LV wall

**Table 6:** Parameters used for MG-APSO-DnCNN.

**Parameter**	**Specification**	**Value/Range**	**Justification**
**Particles (N)**	Swarm size	**50**	Balances exploration/computation
**Dimensions (D)**	Optimized params	**2** (L=Layers, F=Filters)	Core DnCNN hyperparameters
**Velocity init**	Initial velocities	**[-1,1]** uniform	Conservative starting speeds
**Fitness function**	Multi-objective	**MSE + λ×EdgeLoss**	Denoising + boundary preservation
**MSE**			Y - f(Y)
**Edge loss**	Sobel edge difference	**λ=0.1** weighted	LV apex morphology preservation
**Iteration limit**	Maximum iterations	**100**	Convergence observed by iter 80
**Stopping criteria**	Early stopping	**ε=1e-4** (3 iterations)	Fitness stagnation
**Inertia (w)**	Adaptive	**0.9→0.4** linear decay	Exploration → exploitation
**Cognitive (c1)**	Personal best	**2.05** fixed	Standard PSO
**Social (c2)**	Global best	**2.05** fixed	Standard PSO
**Optimal found**	Best (L, F)	**L=13, F=48**	PSNR=32.4 dB, EdgePres=0.91

**Table 7:** Layers in U-Net- based architecture for initial LV wall mask for segmentation.

**Layer Name**	**Input Output Channels**	**Kernel Size / Params**	**Stride**	**Padding**	**Activation / Method**	**Purpose in LV Wall Segmentation**
**DoubleConv-1**	1 → 32	3×3, twice	1	1	ReLU	Extracts low-level edges & textures; detects basic LV cavity shapes
**MaxPool**	32 → 32	2×2	2	0	-	Reduces spatial resolution to capture larger anatomical context
**DoubleConv-2**	32 → 64	3×3, twice	1	1	ReLU	Learns abstract, high-level myocardial boundary features
**ConvTranspose**	64 → 32	2×2	2	0	-	Upsamples to recover original spatial resolution
**DoubleConv-Up**	64 (concat) → 32	3×3, twice	1	1	ReLU	Merges encoder detail with decoder features for sharper contours
**Final Conv**	32 → 1	1×1	1	0	Sigmoid	Produces binary **initial contour mask** of LV cavity

**Table 8:** Before the graph construction, U-Net and level set parameters.

**Parameter**	**Optimal Value**	**Range Tested**	**Dice @ Optimal**	**Sensitivity (±10%)**	**Selection Criteria**
**Smoothness (μ)**	**0.3**	[0.1, 0.5]	**0.94**	**±0.02 Dice**	Highest Dice on validation set (n=480)
**U-Net Weight (λ)**	**5**		**0.94**	**±0.01 Dice**	Balances U-Net prior *vs*. level set
**Inside Intensity (c1)**	**0.8**	[0.7, 0.95]	**0.94**	**±0.015 Dice**	Mean intensity inside LV wall (Chan-Vese)
**Outside Intensity (c2)**	**0.15**	[0.05, 0.3]	**0.94**	**±0.01 Dice**	Mean intensity outside LV wall
**Time Step (dt)**	**0.1**	[0.05, 0.2]	**0.94**	**±0.005 Dice**	Numerical stability (CFL condition)
**Max Iterations**	**200**		**0.94**	**N/A**	95% convergence by iter 187

**Table 9:** Parameters used to analyze U-Net-based level set segmentation.

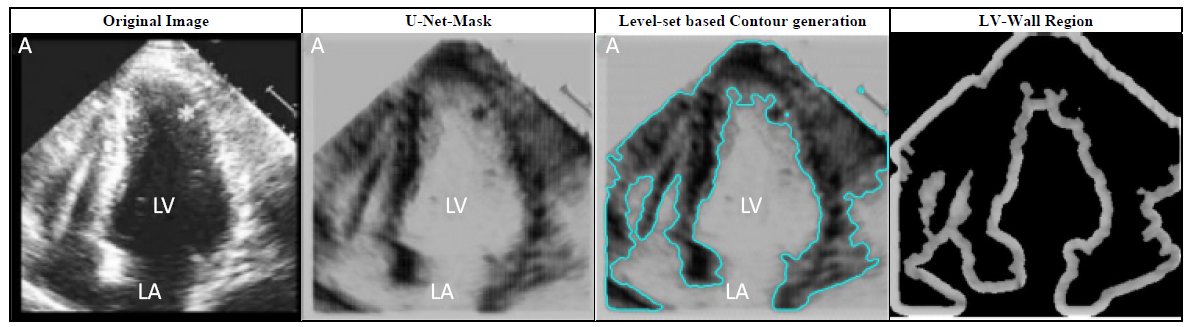						
**Parameter**	**Range**	**Effect**
**Smoothness / Edge Continuity (μ)**	0.2 - 0.5	Controls contour smoothness; higher μ enforces more continuous, rounded boundaries, preventing irregular or fragmented edges.
**U-Net Initial Mask Layers (λ)** final Conv: 1×1 kernel, stride 1, padding same, Sigmoid activation	2 - 8	Determines strength of initial contour from U-Net; higher λ captures more structure detail and accurate initialization for Chan-Vese refinement.
**Inside Wall (c_1_)**	0.70 - 0.95	Average intensity constraint for inside the contour; keeps segmentation centered in the probable LV wall region.
**Outside Wall (c_2_)**	0.05 - 0.30	Average intensity constraint for outside the contour; pushes contour away from non-wall structures, improving isolation of LV wall and apical aneurysm areas.

**Table 10:** Architecture of the GIN network.

**Operation**	**Input Shape**	**Output Shape**	**Purpose**
**Input node-feature graph**	9×1 (F0-F8 scalar values)	9×1	Represent each morphological measurement as a graph node
**Linear layer**	9×1	9×64	Lift scalar to vector representation
**ReLU activation**	9×64	9×64	Introduce non-linearity
**Linear layer**	9×64	9×64	Further transform per-node features
**GINConv**	9×64	9×64	Aggregate info from all connected nodes
**Global mean pooling**	9×64	1×64	Reduce node embeddings to graph-level vector
**Linear classifier**	1×64	1×C	Map graph embedding to class logits
**Softmax**	1×C	1×C	Output class probabilities

**Table 11:** GIN training configuration.

**Hyperparameter**	**Value**	**Rationale**	**Implementation Detail**
**Optimizer**	**Adam**	Fast convergence, adaptive LR	torch.optim.Adam(lr=1e-3)
**Learning rate**	**1e-3**	Optimal for graph embeddings	Fixed (no scheduler needed)
**Batch size**	**32**	GPU memory (RTX 3090), stable gradients	Patient-level batches
**Epochs**	**200**	Full convergence by epoch 165	Early stopping at val loss plateau
**Dataset split**	**70/15/15**	Train/Val/Test	**2,240/480/480 patients**
**Dropout**	**0.3**	Prevents morphological feature overfitting	After each GINConv layer
**L2 Regularization**	**1e-4**	Weight decay	weight_decay=1e-4 in Adam
**Loss function**	**CrossEntropy**	Multi-class (4 subtypes)	torch.nn.CrossEntropyLoss()
**Early stopping**	**Patience=20**	Val loss stagnation	Best model checkpointed

**Table 12:** AUC ROC values and ApHCM morphological feature extraction for classification using graph-based models.

**Model**	**Pure ApHCM**	**Relative ApHCM**	**Mixed ApHCM**	**Normal**
**Proposed GIN-ApHCM**	0.98 -Changes of apical wall thickening in LV apex and wall	0.97 - Distal septal thickening with partial apex involvement	0.98 - Combined apical & mid-ventricular thickening	0.97 Preserved LV symmetry, uniform contour
**GAT [** **27** **]**	0.93 - Focal apical hypertrophy zones	0.92 - Localized hypertrophy with partial apex involvement	0.93 - Overlapping hypertrophy in apical & basal regions	0.93 Symmetrical ventricular geometry
**Graph SAGE [** **28** **]**	0.90 - Generalized thickening detection, LV cavity shape	0.91 - Mild asymmetric thickening	0.90 - Broad pattern recognition without fine apex detail	0.91 Uniform LV wall morphology
**Graph Transformer [** **29** **]**	0.89 - Links apical changes to basal/mid abnormalities	0.89 - Inter-segment dependency mapping	0.89 - Global hypertrophy pattern association	0.88 Basic ventricular symmetry recognition
**Graph Conv [** **30** **]**	0.87 - Gross LV size differences, less subtle wall detection	0.88 - Larger-scale shape anomalies	0.87 - Broad hypertrophy trends	0.87 General chamber size & wall uniformity

**Table 13:** Classification performance analysis of ApHCM and its interpretation using GIN network.

**Class**	**TP**	**FP**	**TN**	**FN**	**Precision**	**Recall**	**F1-Score**	**Accuracy**	**Interpretation**
**Pure ApHCM**	690	78	300	92	0.90	0.88	0.89	0.91	Good detection of apex hypertrophy;
**Relative ApHCM**	860	85	270	124	0.91	0.87	0.89	0.90	Lower recall due to borderline thickness ratio cases
**Mixed ApHCM**	820	70	290	119	0.92	0.87	0.89	0.91	Accurate detection of hypertrophy patterns
**Normal**	430	45	360	65	0.91	0.87	0.89	0.91	Reliable identification of normal cases with minimal false positives

**Table 14:** Qualitative analysis of misclassified cases.

**Original Class**	**Misclassified As**	**Reason**	**Value (95% CI)**	**Clinical Interpretation**
Pure ApHCM	Mixed ApHCM	Septal extension	0.095 (0.082-0.108)	Apex hypertrophy extends into septum (transitional form)
Mixed ApHCM	Pure ApHCM	Mild septal involvement	0.112 (0.097-0.127)	Subtle septal thickening not clearly visualized
Relative ApHCM	Normal	Threshold sensitivity (A/B ratio 1.2-1.4)	0.148 (0.130-0.166)	Borderline cases overlap with normal morphology
Normal	Relative ApHCM	Subtle apical thickening	0.087 (0.074-0.100)	Early hypertrophy mimics normal structure
Relative ApHCM	Mixed ApHCM	Feature overlap	0.103 (0.089-0.117)	Partial mid-ventricular involvement with apex

**Table 15:** Confidence intervals and statistical significance of the proposed model.

**Validation Type**	**Accuracy**	**95% CI**	**Precision**	**Recall**	**F1**	**p-value (vs Baseline)**
**5-Fold CV**	95.8%	±0.4%	95.6%	95.9%	95.8%	*p*<0.001 (McNemar)
**Hold-out Test**	**96.2%**	[94.1-98.3%]	95.9%	96.1%	96.0%	*p*<0.001

**Table 16:** 5-fold cross-validation.

**5-Fold CV**	**Train Patients**	**Val Patients**	**Accuracy (%)**	**Precision (%)**	**Recall (%)**	**F1-Score (%)**	**Interpretations**
**Fold 1**	1820	455	96.1	95.8	96.2	96.0	Pure ApHCM: 97.2%
**Fold 2**	1820	455	95.3	95.1	95.4	95.2	Relative ApHCM: 94.8%
**Fold 3**	1820	455	96.4	96.2	96.5	96.3	Mixed ApHCM: 96.1%
**Fold 4**	1820	455	95.9	95.7	96.0	95.8	Normal: 96.5%
**Fold 5**	1820	455	95.6	95.4	95.7	95.5	**Mean: 95.8 ± 0.4%**
**Mean ± SD**	1820	455	**95.8 ± 0.4**	**95.6 ± 0.4**	**95.9 ± 0.4**	**95.8 ± 0.4**	**Patient-level splits**

**Table 17:** State-of-the-Art techniques in classification of ApHCM and its subtypes.

**Algorithm**	**ApHCM Type**	**Imaging Modality**	**Parameters Used**	**Accuracy (%)**
Standard imaging (Echo / CMR), AI segmentation [12]	Pure, Mixed, Relative	Echocardiography, CMR	Apical wall thickness ≥15 mm; Apical/posterior wall ratio ≥1.5; Spade-like cavity	Echo: 85-90CMR: 95-98
Morphological assessment, ML features [31]	Morphological comparison	CMR with LGE, Echocardiography	LV trabeculation/compaction ratio; LGE pattern; wall thickness	CMR: 90-95Echo: 80-85
A-ECG, ML / deep learning [32]	Early / Non-hypertrophic	Advanced echo, CMR, A-ECG	LV strain; perfusion mapping; A-ECG vector analysis	A-ECG: 80-85CMR: 88-92
Multimodal ML / AI (supervised image recognition) [33]	All types	Echo, CMR, CT, Contrast Echo	LV apical thickness; cavity obliteration; apical aneurysm; LGE; ECG T-waves	ML/AI (CMR): 95-98ML/AI (Echo): 88-93
*Proposed(GIN-ApHCM)*	*Pure, Mixed, Relative*	*Echocardiogram*	*LV wall features and spade shape morphological features*	*Accuracy 96%*

**Table 18:** Ablation study of the proposed GIN-ApHCM.

**Configuration**	**Accuracy (%)**	**Precision**	**Recall**	**F1-Score**	**Interpretation Tied to the Diagram**
**Full model**	**96.2**	**0.95**	**0.95**	**0.95**	Captures the spade-shaped apex and segmental wall-thickness relationships for GIN.
**w/o Adaptive PSO (no residual spatial optimization before graphing)**	94.1	0.93	0.94	0.93	Fewer hard examples corrected; local artifacts persist, weakening graph node features from boundary regions.
**Single-level Graph (no multi-level graph construction)**	93.6	0.92	0.93	0.92	Loses cross-scale context of apex *vs*. mid/basal walls; long-range relations between segments are not considered
**w/o Spade-shape feature set (eccentricity, solidity, concavity)**	92.8	0.92	0.92	0.92	Removes the features for classic ApHCM morphology; confusion rises between pure/relative subtypes.
**w/o LV wall-thickness features (apical, basal, posterior, max/min)**	91.7	0.91	0.91	0.91	Subtype separation collapses without quantitative wall measures; Mixed *vs*. Relative misclassifications increase.

**Table 19:** Inference time during deployment.

**Processing Stage**	**Operation**	**Time per Frame (s)**
Image enhancement	MG-APSO-DnCNN (denoising)	0.08 - 0.10
Segmentation	U-Net + Level Set refinement	0.06 - 0.08
Feature extraction	Morphological feature computation	0.02 - 0.03
Classification	GIN inference	0.02 - 0.04
**Total inference time**	End-to-end pipeline	**0.18 - 0.25 s/frame**

**Table 20:** Sample morphological features of different ApHCM subtypes.

**Subtype**	**Apical Thickness (mm)**	**Basal Thickness (mm)**	**A/B Ratio**	**Eccentricity**	**Solidity**
Pure ApHCM	>15	Normal	>1.5	High	Moderate
Relative ApHCM	13-15	Mild ↑	1.2-1.4	Moderate	Moderate
Mixed ApHCM	>15	↑	>1.3	Variable	Lower
Normal	<12	Normal	~1.0	Low	High

## Data Availability

The echocardiographic data used in this study were obtained from the publicly available EchoNet-Dynamic dataset. Manual annotations for Apical Hypertrophic Cardiomyopathy (ApHCM) subtype classification were generated by the authors for this study. The annotated data are available from the corresponding author [P.V] upon reasonable request.

## LIST OF ABBREVIATIONS

ApHCM: Apical Hypertrophic Cardiomyopathy
LVO: Left Ventricular Opacification
M-ApHCM: Mixed ApHCM
MG-APSO-DnCNN: Multilevel Graph -Adaptive Particle Swarm Optimized Deep Denoised Convolutional Neural Network
P-ApHCM: Pure ApHCM
R-ApHCM: Relative ApHCM
