# Supplementary material for: Deep Learning-based Classification of Apical Hypertrophic Cardiomyopathy using Left Ventricular Opacification and Enhanced LV Apex Imaging
Source: Curr Med Imaging. 2026 Aug 5;22:E15734056450679. doi: 10.2174/0115734056450679260721035709 (PMC13728991; doi:10.2174/0115734056450679260721035709)
Supplement: Supplementary file 1 [file CMIM-22-E15734056450679_SD1.pdf]

1875-6603/26 Send Orders for Reprints to reprints@benthamscience.net

BENTHAM SCIENCE

CrossMark

Current Medical Imaging

Content list available at: https://benthamscience.com/journals/cmir

Supplementary Material

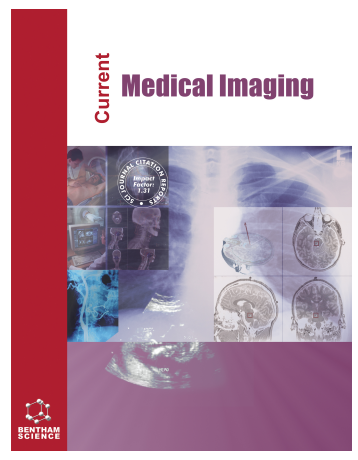

Deep Learning-Based Classification of Apical Hypertrophic Cardiomyopathy Using Left Ventricular Opacification and Enhanced LV Apex Imaging

P. Venkatesan[1,*], A. Rajeswari[2] and N.R. Shanker[3]

[1]Department of Computer Science and Engineering, Vel Tech Multi Tech Dr. Rangarajan Dr. Sakunthala Engineering College, Chennai, India
[2]Department of Computer Science and Engineering, Velammal Engineering College, Chennai, India
[3]Department of Computer Science and Engineering, Sriram Engineering College, Chennai, India

1. HYBRID U-NET AND LEVEL-SET SEGMENTATION

1.1. Initial Segmentation Using U-Net

Given an enhanced echocardiographic image I(x,y), the U-Net model produces a probability map:

$$P(x,y) = \sigma\left(f_U Net\big(I(x,y)\big)\right)$$

A binary segmentation mask is obtained using thresholding:

$$M(x,y) = 1, if P(x,y) > \tau;$$
$$0, otherwise, where \tau = 0.5$$

1.2. Level-Set Initialization

The binary mask generated by U-Net is converted into a signed distance function (SDF) to initialize the level-set contour:

$$\varphi(x,y,0) = +d(x,y) insidethecontour;$$
$$-d(x,y) outsidethecontour$$

1.3. Level-Set Energy Model (Formula 6)

The contour evolution is defined by the following energy minimization model:

$$E(\varphi) = \mu \int ||\nabla\varphi| dxdy + \lambda 1 \int (I - c1)^2 H(\varphi) dxdy$$
$$+ \lambda 2 \int (I - c2)^2 (1 - H(\varphi)) dxdy + \gamma \int \big(P - H(\varphi)\big)^2 dxdy$$

The corresponding evolution equation is:

$$\partial\varphi/\partial t = g(|\nabla I|)[\mu \nabla^2 \varphi - \lambda 1 (I - c1)^2 + \lambda 2 (I - c2)^2]$$

1.4. Contour Refinement Iterations

The level-set evolution is performed iteratively for 50–100 iterations until convergence:

$$|\varphi(t+1) - \varphi(t)| < \varepsilon, where \varepsilon = 10^{-4}$$

1.5. Handling Weak Edge Regions

To address weak-edge regions in echocardiographic images, the following strategies are used:

1. Edge-stopping function: $g(|\nabla I|) = 1/(1 + |\nabla I|^2)$
2. U-Net probability map as shape prior
3. Curvature regularization term: $\mu \int |\nabla\varphi| \, dxdy$

1.6. Final Segmentation

The final LV wall segmentation is obtained after convergence of the level-set function:

$$S(x,y) = 1 if \varphi(x,y) \geq 0; 0 if \varphi(x,y) < 0$$

This hybrid framework combines deep learning-based localization (U-Net) with geometric contour refinement (level-set), enabling accurate LV wall segmentation even in low-contrast echocardiographic regions.

2. FEATURE NORMALIZATION AND GRAPH CONSTRUCTION FOR GIN-BASED ApHCM CLASSIFICATION

2.1. Feature Normalization

In the proposed framework, nine morphological features are extracted from the segmented left ventricular (LV) region of echocardiographic images. These features include eccentricity, solidity, concavity, maximal wall thickness, minimal wall thickness, apical wall thickness, basal wall thickness, posterior wall thickness, and the apex-to-basal thickness ratio. Because these features have different ranges and physical units (e.g., geometric features in the range 0–1 and wall thickness measurements in millimeters), feature normalization is required before training the Graph

DOI: 10.2174/0115734056450679260721035709, 2026, *22*, e15734056450679

Isomorphism Network (GIN).

The extracted feature vector is defined as:

$$X = [x1, x2, x3, x4, x5, x6, x7, x8, x9]$$

Z-score normalization is applied to remove scale differences between features:

$$x_{i_n}orm = (x_i - \mu_i)/\sigma_i$$

where x_i is the original feature value, μ_i is the mean of feature i in the training dataset, and σ_i is the standard deviation of feature i.

2.2. Sample Feature Normalization Example

Example raw morphological features extracted from an LV region:

Feature	Symbol	Raw Value
Eccentricity	x1	0.71
Solidity	x2	0.89
Concavity	x3	0.36
Max Wall Thickness (mm)	x4	18.2
Min Wall Thickness (mm)	x5	8.4
Apical Wall Thickness (mm)	x6	16.5
Basal Wall Thickness (mm)	x7	10.3
Posterior Wall Thickness (mm)	x8	9.8
Apex/Basal Ratio	x9	1.60

Example dataset statistics used for normalization:

Feature	Mean (μ)	Std Dev (σ)
Eccentricity	0.65	0.10
Solidity	0.87	0.05
Concavity	0.40	0.12
Max Wall Thickness	16.0	3.0
Min Wall Thickness	9.0	2.5
Apical Thickness	14.5	2.8
Basal Thickness	11.0	2.1
Posterior Thickness	10.0	1.9
Apex/Basal Ratio	1.35	0.30

Example normalization calculation:

For Max Wall Thickness:

x4_norm = (18.2 − 16.0) / 3.0 = 0.73

For Eccentricity:

x1_norm = (0.71 − 0.65) / 0.10 = 0.60

The normalized feature vector becomes:

X_norm = [0.60, 0.40, -0.33, 0.73, -0.24, 0.71, -0.33, -0.11, 0.83]

2.3. Graph Construction Using GIN

After normalization, a graph is constructed where each LV region is represented as a node and edges represent spatial adjacency between regions.

Graph representation:

G = (V, E)

Example node set:

V = {v1, v2, v3, v4, v5}

Each node contains the normalized feature vector X_norm as node attributes.

Example edge connections:

E = {(v1,v2), (v1,v3), (v2,v4), (v3,v5)}

2.4. Adjacency Matrix Representation

The graph connectivity can be represented using an adjacency matrix A where A(i,j) = 1 if nodes i and j are connected.

[0 1 1 0 0]

[1 0 0 1 0]

[1 0 0 0 1]

[0 1 0 0 0]

[0 0 1 0 0]

2.5. GIN Message Passing

The Graph Isomorphism Network updates node embeddings using neighborhood aggregation:

$$h\_v\hat{}(k) = MLP\hat{}(k)\ ((1 + \varepsilon) * h\_v\hat{}(k-1) + \Sigma\ h\_u\hat{}(k-1))$$

After multiple layers, node embeddings are aggregated to produce the graph-level representation:

h_G = Σ h_v

This representation captures structural relationships between LV regions and is used to classify Apical Hypertrophic Cardiomyopathy subtypes.

3. MATHEMATICAL FORMULATION OF WEISFEILER–LEHMAN BASED GRAPH ISOMORPHISM NETWORK FOR ApHCM ANALYSIS

3.1. Graph Representation of Left Ventricular Regions

The segmented left ventricular (LV) wall is represented as a graph G = (V, E), where V denotes the set of nodes representing myocardial regions (apex, mid-ventricular, and basal segments), and E denotes edges describing spatial adjacency between regions.

Graph definition:

$$G = (V, E)$$

Node set:

$$V = v1, v2, v3, \ldots, vn$$

Edge set:

$$E = (vi, vj) | regionsiandjarespatiallyadjacent$$

Each node vi contains a normalized morphological feature vector Xi consisting of nine features extracted from the LV region.

Node feature vector:

$$Xi = [x1, x2, x3, \dots, x9]$$

3.2. Weisfeiler–Lehman (WL) Feature Aggregation

The Graph Isomorphism Network follows the Weisfeiler–Lehman (WL) graph isomorphism test principle. In the WL framework, node labels are iteratively updated by aggregating the labels of neighboring nodes.

WL label update rule:

$$label\ of\ node\ v(k) \ = HASH(\ label\ of\ node\ v(k-1)\ ,\ \{\ label\ of\ node\ u(k-1)\ |\ u \in\ N(v)\ \}\ )$$

where l_v^(k) represents the label of node v at iteration k and N(v) represents the set of neighboring nodes.

3.3. GIN Node Embedding Update

The GIN architecture replaces the hashing function used in the WL test with a learnable multilayer perceptron (MLP). Node embeddings are updated as follows:

$$Embedding\ node(k) \\ = MLP\hat{\ }k)\ (\ (1\ +\ \varepsilon)\ *\ Embedding\ node\ v(k-1) \\ +\ \Sigma\ Embedding\ node\ u(k-1)\ )$$

where Embedding node (k) denotes the node embedding of node v at layer k, ε is a learnable parameter controlling the contribution of the node's own features, and the summation term aggregates embeddings from neighboring nodes.

3.4. Structural Relationship Learning Between Apex and Ventricular Walls

In the myocardial graph, nodes correspond to anatomical regions such as the apex, mid-ventricular wall, and basal wall. Through iterative neighborhood aggregation, information from neighboring ventricular regions propagates across the graph.

For example, the embedding of the apical node v_apex is updated as:

$$h_{apex}(k) = MLP(k)\left((1+\varepsilon) * h_{apex}(k-1) + \Sigma h_{mid}(k-1)\right)$$

This aggregation allows the model to capture morphological relationships between the LV apex and adjacent ventricular segments.

3.5. Graph-Level Representation

After K GIN layers, node embeddings are aggregated to obtain a graph-level representation describing the overall LV morphology.

Graph embedding:

$$h_G = \Sigma h_v$$

The graph embedding h_G captures structural relationships among LV regions and is used for classification of Apical Hypertrophic Cardiomyopathy subtypes.

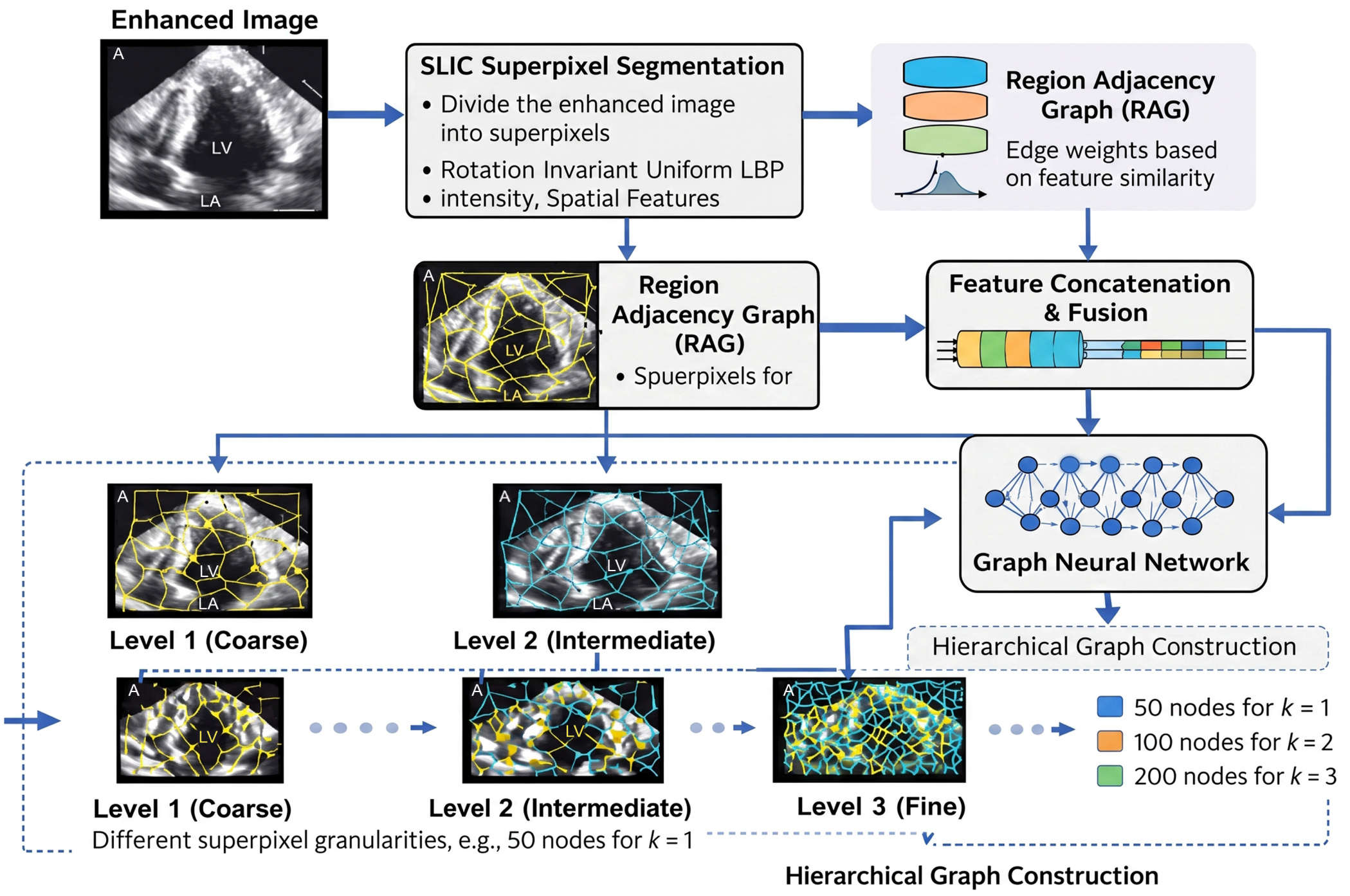

© 2026 The Author(s). Published by Bentham Science Publisher.

This is an open access article distributed under the terms of the Creative Commons Attribution 4.0 International Public License (CC-BY 4.0), a copy of which is available at: https://creativecommons.org/licenses/by/4.0/legalcode. This license permits unrestricted use, distribution, and reproduction in any medium, provided the original author and source are credited.

HOW TO CITE:
Venkatesan P, Rajeswari A, Shanker N. Deep Learning-Based Classification of Apical Hypertrophic Cardiomyopathy Using Left Ventricular Opacification and Enhanced LV Apex Imaging. Curr Med Imaging, 2026; 22: e15734056450679.
http://dx.doi.org/10.2174/0115734056450679260721035709
